# Change in the association between coffee intake and ischemic heart disease in an international ecological study from 1990 to 2018

**DOI:** 10.1038/s41598-022-15611-x

**Published:** 2022-07-05

**Authors:** Yoshiro Shirai, Tomoko Imai, Ayako Sezaki, Keiko Miyamoto, Fumiya Kawase, Chisato Abe, Masayo Sanada, Ayaka Inden, Takumi Kato, Norie Suzuki-Sugihara, Hiroshi Shimokata

**Affiliations:** 1 Department of Food and Nutritional Environment, Kinjo Gakuin University, Aichi, Japan; 2 Department of Food Science and Nutrition, Doshisha Women’s College of Liberal Arts, Kyoto, Japan; 3 Graduate School of Nutritional Science, Nagoya University of Arts and Sciences, Aichi, Japan; 4 Department of Food Science and Human Nutrition, Ryukoku University, Shiga, Japan; 5 Department of Nursing, Nagoya University of Arts and Sciences, Aichi, Japan; 6 Department of Nutrition, Asuke Hospital Aichi Prefectural Welfare Federation of Agricultural Cooperatives, Aichi, Japan; 7 Department of Food and Nutrition, Tsu City College, Mie, Japan; 8 Hamamatsu University Hospital, Shizuoka, Japan; 9 Japanese Red Cross Aichi Medical Centre Nagoya Daini Hospital, Aichi, Japan; 10 Faculty of Core Research, Ochanomizu University, Tokyo, Japan

**Keywords:** Cardiovascular diseases, Epidemiology, Lifestyle modification, Nutrition

## Abstract

In previous observational studies, the association between coffee intake and risk of cardiovascular disease has reversed from positive to negative over time. This long-term international ecological study examined whether the association between coffee intake and mortality and incidence rates of ischemic heart disease (IHD) changed between 1990 and 2018 using multiple coherent data. We obtained data on coffee intake per capita, IHD mortality and incidence rates per 100,000 population, and socioeconomic and lifestyle indicators for each country from various publicly available databases. We integrated and analyzed data from 147 countries with populations of ≥ 1 million. We employed a linear mixed model analysis to assess the association between coffee intake and IHD mortality and incidence rates by year. The mean global coffee intake increased (p < 0.001), whereas IHD mortality (p < 0.001) and incidence (p = 0.073) decreased. In all models, the interaction between coffee intake and year showed a significant inverse association for IHD mortality and incidence rates (p < 0.001 for all). The country-level association between coffee intake and IHD mortality and incidence rates between 1990 and 2018 was stronger in the negative direction.

## Introduction

Coffee is the most widely consumed beverage worldwide. Its public health importance led to studies of its association with cardiovascular health. Basic and clinical research has shown that caffeine and polyphenols (especially chlorogenic acid), which are typical constituents of coffee, are linked to cardiovascular health^1^. An observational study conducted in 1963 was the first to report an association between coffee intake and a higher risk of developing coronary heart disease^2^. Since then, several studies have reported on the issue, but the results have been inconsistent and debatable whether coffee intake is associated with a higher, lower, or no risk of death from cardiovascular disease (CVD), including ischemic heart disease (IHD)^3–13^.

In previous studies, the observed association between coffee intake and risk of heart disease has changed over time. A recent meta-analysis of observational studies reported a preventive association between coffee intake and CVD risk. However, when the analysis included only articles published before 2000, no significant association was observed^14^. Observational studies published in 1976, 1986, and 1987 reported an association of higher IHD risk with coffee intake^5,7,8^, those published in 1990 reported both a higher IHD risk and no association^4,12^, those published between 1992 and 2008 reported no association^6,9,10,13,15^, and those published in 2011, 2012, 2017, and 2020 reported a preventive association^3,11,16,17^. These findings indicate that the association between coffee intake and IHD risk might have changed between pre-1990 and recent times. The change in the results from these reports may be due to environmental factors associated with the changing times (e.g. coffee brewing habits, diet, lifestyle, advances in medicine, and drug treatment) or research-related factors, such as methodologies including dietary surveys, statistical analyses, and sampling and publication bias.

Disease is caused by multiple causal mechanisms, some of which may operate in concert with component causes in complex ways. Furthermore, the disease burden in a population caused by one factor varies from population to population and from era to era as the distribution of other diseases changes^18^. Diet is one of the component causes, and one of the key issues in nutritional epidemiology appears to be the investigation of whether the relationship between dietary intake and disease risk changes over time.

Therefore, to clarify whether the association between coffee intake and IHD mortality and incidence rates at the country-level has changed over time (1990–2018), we conducted a longitudinal ecological study using an international database with multiple coherent data.

## Methods

### Age-standardized mortality and incidence rates of ischemic heart disease

We obtained the annual age-standardized IHD mortality and incidence rates per 100,000 population for each country from 1990 to 2018 from the Global Burden of Disease (GBD) Study 2019^19^. The GBD is a comprehensive programme of global and regional burden studies conducted by the University of Washington’s Institute for Health Metrics and Evaluation as an international collaboration of more than 145 countries^20^. The estimates of the GBD adhere to the Guidelines for Accurate and Transparent Health Estimates Reporting standards developed by the World Health Organization and other organizations.

### Coffee intake

We obtained coffee intake data (cups/day/population) (1 cup = 8 oz) for each country in 1990, 1995, 2000, 2005, 2010, 2015, and 2018 from the Global Dietary Database (GDD)^21^. The GDD is an ongoing collaborative effort to produce the most reliable estimates of worldwide dietary intake to inform research and policymaking on health and nutrition worldwide. Data from the GDD are employed to estimate individual food and nutrient intake worldwide by country, year, sex, etc. The GDD has identified and obtained 1634 eligible survey years of data from public and private sources. Information about handling cases where country-representative surveys are unavailable as well as coding methods and the GDD 2018 prediction model and its validation are described elsewhere^22^.

### Coffee supply

Information on coffee supplies in each country was obtained from the food balance sheet published by the United Nations Food and Agriculture Organization (FAO). The data are available from the FAO Statistics Division database (FAOSTAT), which provides annual data on more than 245 countries and territories^23^. The data are calculated based on various statistics and sources from each country. Currently, data are available from 1961 to 2018. Given that the methodology for estimating the data has changed since 2014, we used data from 1990 (when GBD data were made available) to 2013 (before the change in the estimating methodology) to determine the mean coffee supply (g/day/capita) by country. Further details can be found elsewhere^24^.

### Socioeconomic and lifestyle indicators

Multiple socioeconomic and lifestyle factors are associated with IHD mortality and incidence. To adjust for the association of these factors, we obtained covariates that might correlate to IHD. For the socioeconomic indicators, we obtained data on the gross domestic product (GDP) per capita (US $1000/capita), aging rate (percentage of the population aged ≥ 65 years), and total population by country from the World Bank database from 1990 to 2019^25^.

We obtained lifestyle factors from the GBD covariate database from 1990 to 2019, which included total energy intake (kcal/day/population), mean age-standardized alcohol consumption (g/day/population), age-standardized cigarette smoking rates (%), age-standardized physical activity (1000 metabolic equivalents-min/week), mean body mass index (BMI) for individuals aged > 20 years (kg/m^2^), age-standardized mean systolic blood pressure (SBP) (mmHg) and age-standardized mean low-density lipoprotein cholesterol level (LDL-C) (converting mmol to mg/dL by dividing by 0.02586)^26^. We also obtained the alcohol supply (grams of ethanol/day/capita) and energy supply (kcal/day/capita) by country from FAOSTAT.

Since this study was conducted on a country-by-country basis, the age and sex of the individuals could not be included. For age, however, we used the aging rate as a covariate. The sex distribution was nearly similar in all countries, and when compared across countries, there was little bias due to differences in sex distribution. To account for various regional differences, such as cultural and climatic differences, we also used the “Super Regions” as covariates, which are the seven regions of the GBD’s country classification: Central Europe, Eastern Europe, and Central Asia; Latin America and Caribbean; North Africa and Middle East; South Asia; Southeast Asia, East Asia, and Oceania; Sub-Saharan Africa; and High-income.

### Statistical analysis

We used various statistical data from 1990 to 2018 in which no variables were missing. We limited our analysis to countries with populations of ≥ 1 million. Countries with smaller populations often do not have their own statistical systems, year-to-year variations in statistical values can be large, and outliers can significantly impact the overall results. For our analysis, we included 147 countries for which all data were available.

We examined the distribution and change over time in IHD mortality and incidence rates, coffee intake, and socioeconomic and lifestyle indicators. We assessed the trend of the mean values of each variable in 1990 (the first year of the analysis), 2000 (the early middle year), 2010 (the late middle year), and 2018 (the last year) using a general linear model. We divided the countries based on the Super Regions; calculated the population-weighted mean values for coffee intake and IHD mortality and incidence rates by region for each year; and plotted them using the locally estimated scatterplot smoothing method.

To examine the association between coffee intake and IHD mortality and incidence rates and the changes in the association by year, we conducted a linear mixed model analysis using each country’s IHD mortality and incidence rates over 28 years from 1990 to 2018 as the dependent variables. Coffee intake, year, and the interaction term of coffee intake and year were used as the independent variables. No covariates were added to Model 1, GDP was a covariate in Model 2, and GDP, energy intake, cigarette smoking rate, physical activity, aging rate, and alcohol consumption were covariates in Model 3. All independent variables centered on the grand mean. The random effects in the mixed model were the intercept and slope of the year for each country. We also specified a composite symmetric structure for the covariance matrix for each country and year. The fitting of the model was performed by maximizing the log-likelihood.

For the sensitivity analyses, we further adjusted for BMI, SBP, LDL-C, and Super Regions to validate the robustness of the results. For all of the above analyses over 23 years from 1990 to 2013, we changed the independent variable from the GDD’s coffee intake to the FAOSTAT’s coffee supply and the covariate from the GBD’s energy intake and alcohol consumption to the FAOSTAT’s energy supply (kcal/day/capita) and alcohol supply (ethanol g/day/capita). In addition, stratified analyses were performed for 71 high GDP countries and 72 low GDP countries, separated by the median GDP in 2015.

We used R 4.0.5 for the analyses^27^ and p-values < 0.05 were considered statistically significant. The generalized linear mixed-effects models were fitted using the “lme” function of the “nlme” package^28^.

### Ethical consideration

This study was conducted in compliance with the Declaration of Helsinki. Only publicly available data was used in this study and no personal information was handled.

## Results

Table 1 shows the means and standard deviations of the coffee intake, IHD mortality and incidence rates, and socioeconomic and lifestyle indicators among the countries. The mean coffee intake increased between 1990 and 2018 (p < 0.001), whereas the mean IHD mortality rate and incidence rate decreased from 1990 to 2018 (p < 0.001 and p = 0.073, respectively). The mean GDP, aging rate, total energy intake, BMI, and SBP showed significant upward trends (p < 0.001 for all), whereas the mean cigarette smoking rate showed an inverse trend (p < 0.001).

**Table 1:** Characteristics of the countries by year.

	1990		2000		2010		2018		p trend
	Mean	SD	Mean	SD	Mean	SD	Mean	SD	
n	125		140		146		144		
Coffee intake (cups/day/population)	0.4	0.5	0.5	0.5	0.7	0.6	0.8	0.6	< 0.001
IHD incidence rate (100,000 population)	338.7	182.6	330.6	189.1	321.0	194.7	311.2	187.4	0.073
IHD mortality rate (100,000 population)	187.6	92.9	179.1	108.3	160.9	107.6	147.5	99.5	< 0.001
GDP (US $1000/capita)	5.4	8.4	6.1	9.6	12.6	17.5	14.4	19.2	< 0.001
Population (million population)	40.1	130.0	42.1	141.7	46.0	153.6	50.3	165.2	0.398
Aging rate (%)	6.2	4.2	7.2	4.9	7.9	5.6	9.1	6.6	< 0.001
Cigarette smoking rate (%)	22.2	8.9	20.7	8.8	19.4	8.3	18.5	7.8	< 0.001
Alcohol consumption (grams of ethanol/day/capita)	10.6	7.9	10.5	7.6	10.2	7.6	10.3	7.6	0.537
Physical activity (1000 METs·min/wk)	5.4	1.8	5.5	1.8	5.4	1.8	5.4	1.8	0.988
Total energy intake (1000 kcal/day/capita)	2.5	0.4	2.5	0.4	2.6	0.4	2.7	0.4	< 0.001
BMI (kg/m^2^)	24.0	1.8	24.5	2.0	25.1	2.1	25.6	2.2	< 0.001
SBP (mmHg)	127.7	4.5	128.4	4.4	128.9	4.3	129.4	4.2	< 0.001
LDL-C (mg/dl)	110.2	21.0	111.1	19.2	111.7	17.3	112.7	16.9	0.261

*BMI* body mass index, *GDP* gross domestic product, *IHD* ischemic heart disease, *LDL-C* low-density lipoprotein cholesterol, *MET* metabolic equivalent, *SBP* systolic blood pressure, *SD* standard deviation.

**Figure 1:**
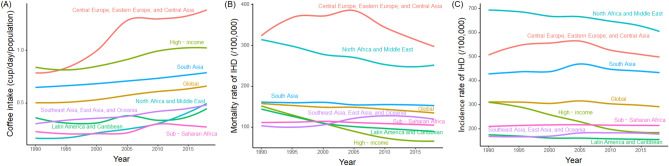
Changes in coffee intake (**A**) and mortality (**B**) and incidence (**C**) rates of ischeemic heart disease (IHD) from 1990 to 2018 globally and for GBD Super Regions.

Figure 1 shows the changes in coffee intake and IHD mortality and incidence rates from 1990 to 2018 globally and for the Super Regions. Although the overall coffee intake increased slowly, it increased significantly in Central Europe, Eastern Europe, and Central Asia between 1995 and 2005. Despite large regional differences in IHD mortality and incidence rates, the overall trend was flat or decreasing.

Tables 2 and 3 show the fixed effects for coffee intake, year, and the interaction between coffee intake and year on the IHD mortality and incidence rates, respectively. In all models, the interaction between coffee intake and year showed a significant inverse association (p < 0.001 for all in Table 2; p < 0.001 for all in Table 3). The significant interaction between coffee intake and year in Model 3 was also shown by further adjusting for BMI, SBP, LDL-C, Super Regions, and all of these in sensitivity analyses. In the order of the adjustment factors listed above, the fixed effects for the interaction between coffee intake and year on the IHD mortality rate were − 1.23, − 1.36, − 1.31, − 1.42, and − 1.25 and those for the IHD incidence rate were − 0.87, − 1.00, − 0.97, − 1.03, and − 0.87 (p < 0.01 for all). In the analysis, when the independent variable was coffee supply (data from FAOSTAT), the associations were similar to coffee intake (Supplementary Tables S1 and S2). In the stratified analyses separated by median GDP, the associations were similar to the main analyses. However, among low GDP countries, the fixed effects for the interaction between coffee intake and year attenuated for mortality (− 0.66, p = 0.14) and incidence (− 0.48, p = 0.18) in Model 3 (Supplementary Tables S3 and S4).

**Table 2:** Fixed effects of coffee intake, year, coffee intake–year interaction, and covariates on the IHD mortality rate per 100,000 population in the three linear mixed-effects models.

	Model 1	Model 2	Model 3
	β (SE)	β (SE)	β (SE)
(Intercept)	173.046 (8.549)***	173.027 (8.418)***	224.033 (15.218)***
Coffee intake	− 10.047 (3.516)**	− 8.417 (3.518)*	− 10.294 (3.387)**
Year	− 1.754 (0.244)***	− 1.449 (0.247)***	− 1.252 (0.314)***
Coffee*Year	− 1.552 (0.259)***	− 1.431 (0.258)***	− 1.375 (0.251)***
GDP		− 0.955 (0.239)***	− 0.582 (0.245)*
Total energy intake			− 80.84 (11.979)***
Cigarette smoking rate			1.523 (0.580)**
Physical activity			− 9.475 (4.999)
Aging rate			4.871 (1.102)***
Alcohol consumption			− 4.941 (1.219)***
AIC	9677.8	9665.8	9589.2
BIC	9721.8	9714.7	9662.4

Model 1: No covariates were adjusted.

Model 2: GDP (US$1000/capita) was adjusted.

Model 3: GDP, total energy intake (1000 kcal/day/capita), cigarette smoking rate (%), physical activity (1000 metabolic equivalents-min/week), aging rate (%), and alcohol consumption (grams of ethanol/day/capita) were adjusted.

*GDP* gross domestic product, *BMI* body mass index, *AIC* Akaike’s information criterion, *BIC* Bayesian information criterion, *SE* standard error.

***p < 0.001, **p < 0.01, *p < 0.05.

**Table 3:** Fixed effects of coffee intake, year, coffee intake–year interaction, and covariates on the IHD incidence rate per 100,000 population in the three linear mixed-effects models.

	Model 1	Model 2	Model 3
	β (SE)	β (SE)	β (SE)
(Intercept)	331.052 (15.772)***	331.074 (15.749)***	400.790 (25.143)***
Coffee intake	− 6.596 (3.093)*	− 5.586 (3.093)	− 6.634 (2.960)*
Year	− 1.149 (0.225)***	− 0.896 (0.227)***	− 0.589 (0.291)*
Coffee*Year	− 1.244 (0.227)***	− 1.164 (0.226)***	− 1.028 (0.219)***
GDP		− 0.789 (0.223)***	− 0.456 (0.219)*
Total energy intake			− 87.179 (11.400)***
Cigarette smoking rate			1.722 (0.550)**
Physical activity			− 30.371 (8.038)***
Aging rate			3.563 (1.095)**
Alcohol consumption			− 6.769 (1.943)***
AIC	9625.3	9616.6	9524.2
BIC	9669.3	9665.4	9597.4

Model 1: No covariates were adjusted.

Model 2: GDP (US$1,000/capita) was adjusted.

Model 3: GDP, total energy intake (1000 kcal/day/capita), cigarette smoking rate (%), physical activity (1000 metabolic equivalents-min/week), aging rate (%), and alcohol consumption (grams of ethanol/day/capita) were adjusted.

*GDP* gross domestic product, *BMI* body mass index, *AIC* Akaike’s information criterion, *BIC* Bayesian information criterion, *SE* standard error.

***p < 0.001, **p < 0.01, *p < 0.05.

**Figure 2:**
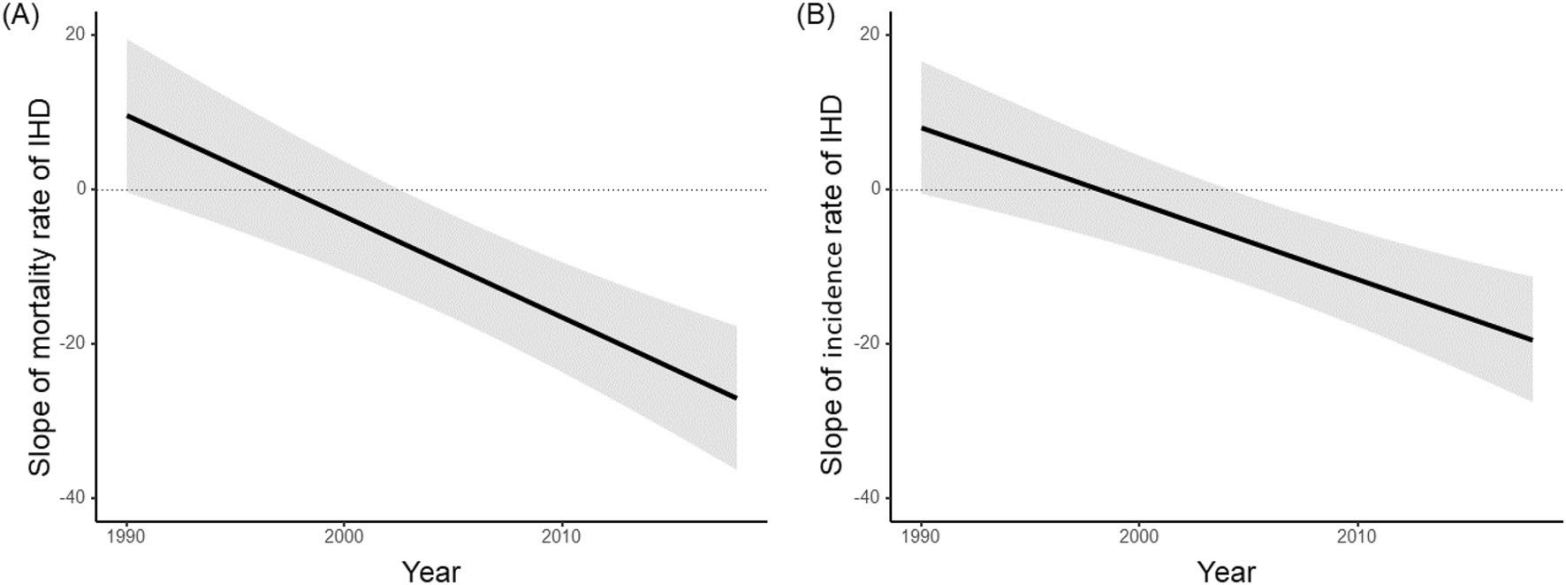
Estimated change in slope and 95% confidence interval of coffee intake to mortality (**A**) and incidence (**B**) rates of ischemic heart disease (IHD) from 1990 to 2018 by mixed-effects model adjusting for covariates.

We examined the associations between coffee intake and IHD mortality and incidence rates by year using the mixed-effects model, adjusting for covariates in Model 3. The estimates of slope and 95% confidence interval between coffee intake and IHD mortality and incidence rates in 1990, 1995, 2000, 2005, 2010, 2015, and 2018 are shown in Fig. 2. The slope of coffee intake on the IHD mortality and incidence rates reversed from positive to negative and lowered over the years. The slope of IHD mortality rate was positive in 1990 (β = 10.5, p = 0.04) to 1992 (β = 7.71, p = 0.01), then unrelated until 2001 and then negative from 2002 (β =  − 6.03, p = 0.08) to 2018 (β =  − 28.03, p < 0.001). The slope of IHD incidence rate was positive in 1990 (β = 8.89, p = 0.04) to 1992 (β = 6.84, p = 0.09), then unrelated until 2003 and negative from 2004 (β =  − 5.50, p = 0.06) to 2018 (β =  − 19.9, p < 0.001).

## Discussion

This study used multiple international databases to ecologically show the country-level association between coffee intake and IHD mortality and incidence rates from 1990 to 2018. The results showed that the association between coffee intake and IHD mortality and incidence rates shifted from a positive association in 1990 to an inverse association by 2018. To our knowledge, this study is the first to report longitudinal changes in the association between coffee and IHD using public data on a global scale.

According to a meta-analysis of the association between coffee intake and CVD risk, possible explanations for the lack of consistency between older and more recent observational studies include differing risks depending on how the coffee is brewed and confounding by cigarette smoking^14^. Regarding coffee brewing methods, a cohort study of approximately 500,000 individuals followed for a mean of 20 years found that CVD mortality was higher in those who consumed unfiltered coffee than those who consumed paper-filtered coffee. The association between unfiltered coffee intake and higher IHD mortality was suggested to be partially mediated by total cholesterol^29^. In the 1980s and 1990s, there was growing interest in the different health effects of unfiltered and paper-filtered coffee, with several studies showing differences. In the 1980s, studies showed that coffee intake was associated with increased serum cholesterol^30–32^. In 1991, two clinical studies reported that components contained in boiled coffee that raise serum cholesterol levels are removed by the use of paper filters^33,34^. In 1994, cafestol and carweol, natural components of coffee, were shown to be associated with the increase in serum total cholesterol levels^35^. Preparation techniques for various coffee brews showed that these components in filtered coffee and instant coffee were extremely low compared with unfiltered coffee^36^. The results of these studies may have encouraged individuals to use paper filters for coffee brewing. Today, paper filter brewing is common in many parts of the world, especially in high-income countries. The number of studies reporting differences in coffee brewing methods in the general population is very limited. To our knowledge, only three studies were published in the 1980s^29,37,38^, covering just Norway and Finland. These three studies had 18,012 (20–59 years), 508,747 (20–79 years), and 5704 (25–64 years) participants, where they found that unfiltered and filtered coffee were consumed at rates of 20% and 59%, 68% and 21.5%, and 24% and 69%, respectively. A substantial number of individuals who consumed boiled coffee might have shifted to filtered coffee in recent years, which might be one of the reasons for the change in the association between coffee consumption and IHD risk.

Regarding confounding by cigarette smoking, a strong relationship between cigarette smoking and coffee consumption has been reported^39^. This relationship may be biased toward the association of coffee intake with higher IHD risk, and previous meta-analysis studies reported that adjustment for cigarette smoking strengthened the inverse association between coffee intake and heart disease risk^14,39^. In the present data, cigarette smoking rates were positively associated with IHD mortality and incidence rates longitudinally. Although the mean cigarette smoking rate in each country decreased by 3.7% between 1990 and 2018, the mean coffee intake in each country doubled. This decrease in smoking rates and increase in coffee intake could be one of the reasons for the change in the association between coffee intake and IHD risk.

As for changes in the social context, the evolution of information technology, which has brought about rapid changes in society since 1995, might have also influenced the association between coffee intake and IHD risk. The Internet has made it easier for people to access various information, increasing accessibility to medical and health information and likely enhancing public health awareness. If individuals follow healthier diets and lifestyle habits, fewer people will develop IHD and die from it. For instance, some people consume coffee with added sugar and milk (including saturated fatty acids). When sugar and saturated fatty acids in coffee are reduced (replaced with starch and polyunsaturated fatty acids), coffee consumption is reportedly more favorable for cardiovascular health^40,41^. With medical and pharmaceutical advances, the number of deaths from IHD is expected to decrease, regardless of coffee consumption. In our study, the difference in IHD mortality rate between 1990 and 2018 (− 40.1) was greater than that in incidence rates (− 27.5), and this difference is probably attributable to improved medical infrastructure, health services, etc. These changes in the social context might have influenced the change in the association between coffee intake and IHD risk.

The limitations of this study include the fact that it is a country-level ecological study, which cannot consider individual age, sex, race, coffee brewing methods and additives, and dietary habits. In addition, the effects of selection bias and unmeasured confounders remain. Therefore, this study cannot explain the causal relationship between coffee intake and IHD mortality and incidence owing to the study design. Nevertheless, it did reveal that the association has changed over time. However, important confounding factors, such as cigarette smoking, alcohol consumption, and physical activity, were considered. Although coffee brewing methods, use of additives, and the level of healthcare provision can vary by region, the results did not change when we included the seven GBD Super Regions in the model. The results were also consistent in the models that included BMI, SBP, LDL-C, or all of these factors associated with IHD mortality and incidence. Furthermore, we analyzed two different databases regarding coffee intake; the GDD, which estimated food intake, and the FAOSTAT (1990–2013), which estimated food supply. In the analyses using each data, the association between coffee intake and IHD and the changes in this association since 1990 were similar. A further limitation is that we were unable to evaluate the relationship before 1990. Although reports indicating the association of coffee intake with increased heart disease risk were mainly from the 1980s, data from this period were unavailable in the present study.

One of the strengths of this long-term ecological study is that the data employed in the analysis were estimated at the national level from multiple databases by researchers unrelated to this research project. The data objectively showed the association between coffee intake and IHD mortality and incidence rates. The results suggest that period-specific associations might have existed; from the association of high IHD risk with coffee intake around 1990 to no association around 2000 and the association of low IHD risk with coffee intake subsequently.

This study suggests that the association between coffee intake and IHD risk may change over time owing to changes in social and environmental factors related to the distribution of the disease and its component causes. We propose that the association between dietary habits and disease risks in a given era (especially the results of older studies) can vary over time.

## Supplementary Information

Supplementary Table S1.

Supplementary Table S2.

Supplementary Table S3.

Supplementary Table S4.

## Data Availability

All data used in this study are available from the organizations listed in the text or from the corresponding author upon reasonable request.
