# Supplementary material for: Change in the association between coffee intake and ischemic heart disease in an international ecological study from 1990 to 2018
Source: Sci Rep. 2022 Jul 5;12:11319. doi: 10.1038/s41598-022-15611-x (PMC9256668; doi:10.1038/s41598-022-15611-x)
Supplement: Supplementary file 1 — Supplementary Table S1. [file 41598_2022_15611_MOESM1_ESM.pdf]

Supplemental table 1. Fixed effects of coffee supply, year, coffee supply-year interaction, and covariates on the **IHD mortality rate per 100,000 population** in the three linear mixed-effects models.

	Model 1			Model 2			Model 3		
	β (SE)			β (SE)			β (SE)		
(Intercept)	177.43	(9.15)	***	177.40	(9.09)	***	177.94	(9.85)	***
Coffee supply	-0.05	(0.13)		0.01	(0.13)		-0.16	(0.13)	
Year (1990 to 2013)	-1.94	(0.31)	***	-1.81	(0.31)	***	-2.30	(0.36)	***
Coffee*Year	-0.11	(0.02)	***	-0.10	(0.02)	***	-0.07	(0.02)	***
GDP				-0.31	(0.09)	***	-0.35	(0.09)	***
Total energy supply							-12.12	(2.74)	***
Cigarette smoking rate							0.06	(0.31)	
Physical activity							12.59	(4.95)	*
Aging rate							9.20	(0.81)	***
Alcohol supply							-0.01	(0.02)	
AIC	25650.1			25643.0			25501.2		
BIC	25704.5			25703.4			25591.9		

GDP: gross domestic product, BMI: body mass index, AIC: Akaike's information criterion, BIC: Bayesian information criterion, SE: standard error

Model 1: No covariates were adjusted.

Model 2: GDP was adjusted.

Model 3: GDP, total energy supply (1,000 kcal/day/capita), cigarette smoking rate (%), physical activity (1,000 metabolic equivalents-min/week), ageing rate (%) and alcohol supply (grams of ethanol/day/capita) were adjusted.

*** $p < 0.001$, ** $p < 0.01$, * $p < 0.05$
