# Supplementary material for: Change in the association between coffee intake and ischemic heart disease in an international ecological study from 1990 to 2018
Source: Sci Rep. 2022 Jul 5;12:11319. doi: 10.1038/s41598-022-15611-x (PMC9256668; doi:10.1038/s41598-022-15611-x)
Supplement: Supplementary file 2 — Supplementary Table S2. [file 41598_2022_15611_MOESM2_ESM.pdf]

Supplemental table 2. Fixed effects of coffee supply, year, coffee supply-year interaction, and covariates on the **IHD incidence rate per 100,000 population** in the three linear mixed-

	Model 1			Model 2			Model 3		
	β (SE)			β (SE)			β (SE)		
(Intercept)	331.56	(16.48)	***	331.54	(16.46)	***	333.21	(18.92)	***
Coffee supply	0.07	(0.10)		0.10	(0.11)		-0.03	(0.10)	
Year (1990 to 2013)	-1.39	(0.27)	***	-1.30	(0.27)	***	-1.55	(0.33)	***
Coffee*Year	-0.07	(0.01)	***	-0.07	(0.01)	***	-0.04	(0.01)	**
GDP				-0.22	(0.07)	**	-0.21	(0.07)	**
Total energy supply							-16.46	(2.09)	***
Cigarette smoking rate							0.32	(0.24)	
Physical activity							37.90	(7.05)	***
Aging rate							8.80	(0.67)	***
Alcohol supply							-0.03	(0.01)	**
AIC	24371.1			24367.0			24083.1		
BIC	24425.5			24427.5			24173.7		

GDP: gross domestic product, BMI: body mass index, AIC: Akaike’s information criterion, BIC: Bayesian information criterion, SE: standard error

Model 1: No covariates were adjusted.

Model 2: GDP was adjusted.

Model 3: GDP, total energy supply (1,000 kcal/day/capita), cigarette smoking rate (%), physical activity (1,000 metabolic equivalents-min/week), aging rate (%) and alcohol supply (grams of ethanol/day/capita) were adjusted.

*** $p < 0.001$, ** $p < 0.01$, * $p < 0.05$
