# Supplementary material for: Change in the association between coffee intake and ischemic heart disease in an international ecological study from 1990 to 2018
Source: Sci Rep. 2022 Jul 5;12:11319. doi: 10.1038/s41598-022-15611-x (PMC9256668; doi:10.1038/s41598-022-15611-x)
Supplement: Supplementary file 3 — Supplementary Table S3. [file 41598_2022_15611_MOESM3_ESM.pdf]

Supplemental table 3. Fixed effects of coffee intake, year, coffee intake-year interaction, and covariates on the **IHD mortality rate per 100,000 population** in stratified analysis by median GDP in 2015.

	Model 1			Model 2			Model 3		
	β (SE)			β (SE)			β (SE)		
**High GDP countries (n=71)**									
(Intercept)	163.12	(10.39)	***	162.74	(9.86)	***	205.98	(16.92)	***
Coffee intake	-6.90	(4.09)		-4.04	(4.03)		-9.77	(4.07)	*
Year (1990 to 2018)	-3.40	(0.24)	***	-2.91	(0.26)	***	-3.22	(0.37)	***
Coffee*Year	-1.08	(0.28)	***	-0.88	(0.27)	**	-1.24	(0.29)	***
GDP				-0.83	(0.21)	***	-0.31	(0.23)	
Total energy intake							-62.39	(15.84)	***
Cigarette smoking rate							0.88	(0.67)	
Physical activity							9.93	(8.09)	
Aging rate							3.81	(1.15)	**
Alcohol consumption							-4.05	(1.27)	**
AIC	4648.4			4638.0			4594.1		
BIC	4686.0			4679.7			4656.5		
**Low GDP countries (n=72)**									
(Intercept)	181.77	(13.94)	***	181.42	(14.04)	***	228.86	(25.28)	***
Coffee intake	-10.29	(5.93)		-6.67	(5.92)		-9.90	(5.69)	
Year (1990 to 2018)	-0.16	(0.37)		0.22	(0.38)		0.74	(0.45)	
Coffee*Year	-1.61	(0.46)	***	-1.19	(0.47)	*	-0.66	(0.45)	
GDP				-6.41	(1.86)	***	-4.46	(2.00)	*
Total energy intake							-92.65	(19.65)	***
Cigarette smoking rate							2.29	(1.02)	*
Physical activity							-22.56	(7.37)	**
Aging rate							9.20	(2.66)	***
Alcohol consumption							-4.82	(2.13)	*
AIC	4851.8			4839.0			4778.0		
BIC	4889.4			4880.8			4840.5		

GDP: gross domestic product, BMI: body mass index, AIC: Akaike's information criterion, BIC: Bayesian information criterion, SE: standard error

Model 1: No covariates were adjusted.

Model 2: GDP (US$1,000/capita) was adjusted.

Model 3: GDP, total energy intake (1,000 kcal/day/capita), cigarette smoking rate (%), physical activity (1,000 metabolic equivalents-min/week), aging rate (%) and alcohol consumption (grams of ethanol/day/capita) were adjusted.

*** $p < 0.001$, ** $p < 0.01$, * $p < 0.05$
