# Supplementary material for: Change in the association between coffee intake and ischemic heart disease in an international ecological study from 1990 to 2018
Source: Sci Rep. 2022 Jul 5;12:11319. doi: 10.1038/s41598-022-15611-x (PMC9256668; doi:10.1038/s41598-022-15611-x)
Supplement: Supplementary file 4 — Supplementary Table S4. [file 41598_2022_15611_MOESM4_ESM.pdf]

Supplemental table 4. Fixed effects of coffee intake, year, coffee intake-year interaction, and covariates on the **IHD incidence rate per 100,000 population** in stratified analysis by median GDP in 2015.

	Model 1			Model 2			Model 3		
	β (SE)			β (SE)			β (SE)		
**High GDP countries (n=71)**									
(Intercept)	330.80	(20.37)	***	330.67	(20.18)	***	371.16	(34.05)	***
Coffee intake	-5.64	(4.20)		-4.65	(4.21)		-8.29	(4.01)	*
Year (1990 to 2018)	-2.51	(0.29)	***	-2.23	(0.32)	***	-2.74	(0.45)	***
Coffee*Year	-0.95	(0.30)	**	-0.90	(0.30)	**	-1.07	(0.30)	***
GDP				-0.48	(0.24)	*	-0.09	(0.23)	
Total energy intake							-70.25	(17.10)	***
Cigarette smoking rate							1.20	(0.71)	
Physical activity							7.17	(15.17)	
Aging rate							6.10	(1.36)	***
Alcohol consumption							-3.81	(2.42)	
AIC	4723.2			4722.3			4672.5		
BIC	4760.8			4764.0			4734.8		
**Low GDP countries (n=72)**									
(Intercept)	327.25	(24.47)	***	326.88	(24.56)	***	436.62	(41.49)	***
Coffee intake	-6.98	(4.81)		-2.81	(4.75)		-4.53	(4.52)	
Year (1990 to 2018)	0.15	(0.30)		0.56	(0.32)		1.16	(0.37)	**
Coffee*Year	-1.27	(0.37)	***	-0.81	(0.37)	*	-0.48	(0.36)	
GDP				-6.94	(1.49)	***	-4.32	(1.62)	**
Total energy intake							-102.00	(16.98)	***
Cigarette smoking rate							1.97	(0.90)	*
Physical activity							-46.87	(11.76)	***
Aging rate							3.86	(2.26)	
Alcohol consumption							-11.06	(3.44)	**
AIC	4759.1			4737.7			4660.8		
BIC	4796.7			4779.5			4723.3		

GDP: gross domestic product, BMI: body mass index, AIC: Akaike's information criterion, BIC: Bayesian information criterion, SE: standard error

Model 1: No covariates were adjusted.

Model 2: GDP (US$1,000/capita) was adjusted.

Model 3: GDP, total energy intake (1,000 kcal/day/capita), cigarette smoking rate (%), physical activity (1,000 metabolic equivalents-min/week), aging rate (%) and alcohol consumption (grams of ethanol/day/capita) were adjusted.

*** $p < 0.001$, ** $p < 0.01$, * $p < 0.05$
